# ENIGMA+: a national, decentralized, remote consent study for clinical data and biospecimen collection in patients with ALK-positive advanced NSCLC

**DOI:** 10.1093/oncolo/oyaf217

**Published:** 2025-07-17

**Authors:** Joyce Liang, Sarah Waliany, Andrew Do, Jennifer L Peterson, Paige Roberts, Elizabeth A Kennedy, Emily S Venanzi, Justin F Gainor, Jessica J Lin

**Affiliations:** Department of Medicine and Cancer Center, Massachusetts General Hospital, Boston, MA, United States; Department of Medicine and Cancer Center, Massachusetts General Hospital, Boston, MA, United States; Department of Medicine and Cancer Center, Massachusetts General Hospital, Boston, MA, United States; Department of Medicine and Cancer Center, Massachusetts General Hospital, Boston, MA, United States; Department of Medicine and Cancer Center, Massachusetts General Hospital, Boston, MA, United States; Department of Medicine and Cancer Center, Massachusetts General Hospital, Boston, MA, United States; ALK Positive, Inc., Atlanta, GA, 30328, United States; Department of Medicine and Cancer Center, Massachusetts General Hospital, Boston, MA, United States; Department of Medicine and Cancer Center, Massachusetts General Hospital, Boston, MA, United States

**Keywords:** ALK, NSCLC, remote consent, patient diversity, resistance

## Abstract

**Objective:**

Despite advances in ALK inhibitors for *ALK* fusion-positive (ALK+) non–small-cell lung cancer (NSCLC), drug resistance remains a challenge. Studies of treatment outcomes and resistance biomarkers are imperative for drug development, yet patient representation can be limited. This study evaluated the feasibility of a decentralized research infrastructure to establish a clinical and biospecimen repository, broadening patient access and inclusion.

**Patients and methods:**

Patients with advanced ALK+ NSCLC across the United States were enrolled through remote informed consent. Clinical history and tumor molecular profiling data were collected at baseline and during remote follow-ups. Archival tumor and saliva biospecimens (for germline sampling) were obtained for analysis.

**Results:**

Of 87 eligible patients, 80 (92%) completed remote consent and enrolled. The clinical data collection rate was 100%, with archival tumor acquired from 80% and saliva samples from 65%. Patients represented 31 states, with 94% residing outside the study center’s state and 90% receiving care elsewhere. Next-generation sequencing was conducted on 55 treatment-naïve and 18 treatment-resistant biopsies, all of whom received at least one prior second-generation ALK inhibitor, and 9 received lorlatinib. *ALK* resistance mutations were identified in 54% of treatment-resistant biopsies; other commonly co-altered genes included *TP53* (18%) and *CDKN2A/B* (16%).

**Conclusions:**

This study highlights the feasibility of a decentralized design to enhance the inclusion of a broader patient population with ALK+ NSCLC. This establishes a scalable framework that may help overcome barriers to patient participation in research, with the goal of improving therapy development and patient outcomes. The Elucidating Novel Immune and Genomic Markers for ALK+ study accrual and analysis continue (NCT04881916).

Implications for Practice Multiple generations of ALK inhibitors are approved for patients with *ALK* fusion-positive (ALK+) lung cancer, but drug resistance inevitably develops, causing disease relapse. Understanding real-world treatment patterns, clinical outcomes, and resistance mechanisms is crucial, yet collecting diverse, comprehensive data remains challenging. ENIGMA+ is an innovative nationwide decentralized study infrastructure that leverages remote patient consenting and participation to expand patient access and inclusion. Findings demonstrate the feasibility of establishing a geographically broad clinical data and biospecimen repository. The ENIGMA+ design offers a scalable approach to broaden research participation, facilitate equitable knowledge generation, and accelerate advances in precision oncology.

## Introduction

Anaplastic lymphoma kinase (*ALK*) gene fusions represent a potent oncogenic driver present in 3%-5% of patients with non–small-cell lung cancer (NSCLC).^1^^,^^2^ Since the initial discovery of the *ALK* fusion oncogene driver in lung cancer in 2007, the development of numerous ALK tyrosine kinase inhibitors (TKIs) has transformed the treatment paradigm for patients diagnosed with advanced or metastatic *ALK* fusion-positive (ALK+) NSCLC, resulting in substantially improved outcomes and prognoses for patients.^2^ Multiple generations of ALK TKIs have been developed—ranging from first-generation TKI crizotinib to second- and third-generation agents, such as alectinib, brigatinib, ensartinib, and lorlatinib—that demonstrated efficacy and tolerability, with variable degrees of central nervous system activity and potency against *ALK* resistance mutations.^3–7^

Despite such significant therapeutic advances, major challenges persist in the management of patients with ALK+ lung cancer. Drug resistance is a fundamental issue that inevitably limits the duration of benefit from even higher-generation ALK inhibitors, and effective therapies for TKI-refractory disease remain an urgent unmet need.^8^ Beyond biological resistance, systemic barriers undermine optimal care, including disparities in access to therapies, clinical trials, and research participation.^9–12^ Patients with ALK+ lung cancer often seek second opinions from major cancer centers to access subspecialized expertise and novel investigational therapies, which may facilitate the development of more personalized treatment plans.^13^ However, many patients face barriers in traveling to a large academic center to seek oncologic care or to participate in research due to physical, psychosocial, or financial burdens, in addition to time constraints.^14–16^ As a result, these patients—and the biology of their cancers—remain underrepresented in clinical trials and in the broader body of cancer research, which may impede adequately informed and broadly applicable therapeutic development.

To address these fundamental challenges and concretely enhance access to research and inclusivity for patients with advanced ALK+ NSCLC, we launched and implemented the “Elucidating Novel Immune and Genomic Markers for ALK+ (ENIGMA+)” study. This study utilizes a fully remote patient enrollment and participation process through remote informed consents, follow-ups, and connection with patients and their primary local treating centers. The primary goal of the study is to establish a comprehensive nationwide clinical data and biospecimen repository for the study of ALK+ lung cancer biology. ENIGMA+ represents an innovative national, decentralized research infrastructure that seeks to bridge the gap in equitable patient representation in research. Here, we report the ENIGMA+ study design and structure as well as the first study analysis.

## Methods

### Patients and study eligibility

This study (NCT04881916), conducted under an Institutional Review Board (IRB)-approved protocol, was led by investigators at the Massachusetts General Hospital (MGH) in collaboration with the Addario Lung Cancer Medical Institute (ALCMI) study team. The study adhered to the Health Insurance Portability and Accountability Act Privacy Rule, ensuring the confidentiality, secure storage, and restricted access of patients’ protected health information. Access to patient data was restricted to authorized personnel, and patient information was de-identified to safeguard participant privacy throughout the study.

Patients were eligible for inclusion if they were aged 18 years or older at the time of consent, had proficiency in English, and had a confirmed diagnosis of advanced stage ALK+ NSCLC. Additionally, patients needed to be willing to share clinical and medical data, including archival tumor tissue samples and saliva. The study was designed with lenient eligibility criteria to maximize patient inclusion and participation.

### Remote consent process

The study utilized a fully remote, IRB-compliant consent process to streamline patient enrollment while enhancing accessibility and inclusivity. Outreach efforts included social media engagement conducted by the ALCMI and MGH study team and collaborative interviews with ALK Positive, a patient-led nonprofit organization dedicated to supporting families and patients affected by ALK+ lung cancer, to broaden study awareness. Patients interested in the study initiated contact with the research team through a form available on the study website.^17^^,^^18^ The ALCMI study team conducted prescreening and informed consent discussions with prospective participants via telephone, ensuring clear understanding of study requirements and objectives. Patients were provided with secure electronic consent forms through the REDCap platform, enabling them to review the documents, ask questions, and electronically sign the forms in a secure environment. These forms included the Informed Consent, Medical Release, and Intake Forms necessary for participation. Patients were informed that the study would involve genomic and germline testing (of biospecimens), and that tests done on this study are only for research. Once consent was obtained, the MGH study team enrolled participants and conducted follow-up to collect medical records and biospecimens.

### Follow-up

From the time of study consent, enrolled patients were followed remotely for up to 24 months, until death, or consent withdrawal, whichever occurred first. Follow-up was conducted at 6, 12, and 24-month post-enrollment. During each follow-up, data were collected through patients or their treating centers on treatment changes, disease status, survival status, and the availability of additional archival tissue samples to ensure precise tracking of each patient’s clinical trajectory, with the ultimate goal of accurately capturing longitudinal treatment outcomes and patient biospecimen availability.

### Clinical data and biospecimen collection

Clinical data and biospecimens were collected at the prespecified time points by the study team in coordination with the patients’ primary treating hospitals. Key clinical data extracted from medical records included demographic characteristics, date of advanced lung cancer diagnosis, sites of metastatic disease, pathology and molecular testing results at initial diagnosis and/or during cancer resistance, targeted and non-targeted systemic treatment history (including the specific treatment received, duration of therapy, and disease progression status), locoregional treatment history (eg, surgery, radiation), and survival status.

Archival tumor tissues were requested and collected if available, with a preference for up to 15 unstained formalin-fixed, paraffin-embedded (FFPE) slides or a single FFPE block. Patients who had at least one archival tumor sample collected were asked to provide saliva samples for paired analyses and to provide germline samples for sequencing analyses. To facilitate this, DNA Genotek saliva kits were shipped directly to participating patients, who self-collected the saliva sample and returned the completed kits by mail to the study team.

All patient data collected during the study were securely stored in a REDCap database, identified by uniquely assigned subject IDs to ensure confidentiality and data integrity.

### Study endpoints

The primary study endpoints were the rates of successful remote consent, medical record collection, and biospecimen acquisition. Secondary endpoints encompassed real-world treatment patterns and efficacy measures, including the duration of therapy and overall survival (OS).

### Statistical analysis

Demographic characteristics were summarized descriptively. The duration of therapy was calculated from “day 0,” defined as the initiation date of therapy to therapy switch or discontinuation. Patients continuing on the therapy of interest at data cut-off were censored at the date of last follow-up. OS was measured from the date of advanced lung cancer diagnosis to the date of death, with living patients censored at the date of last follow-up. OS stratified by initial therapy was measured from the initiation date of therapy to the date of death, with living patients censored at the last follow-up date. Kaplan-Meier method was used to estimate treatment duration and OS, with Cox proportional hazards regression models used to estimate the hazard ratio (HR) comparing treatment duration and OS between subgroups of interest. Statistical analyses and visualizations were conducted using R-Studio, leveraging a suite of specialized pre-built packages, including ggplot2, ComplexHeatmap, swimplot, ggsurvfit, and ggsankey.

The data cut-off point for this analysis was September 1, 2024. The study is ongoing, with participant accrual continuing toward the goal of 100 and with patient follow-up in progress.

## Results

### The study cohort and patient disposition

**Figure 1.**
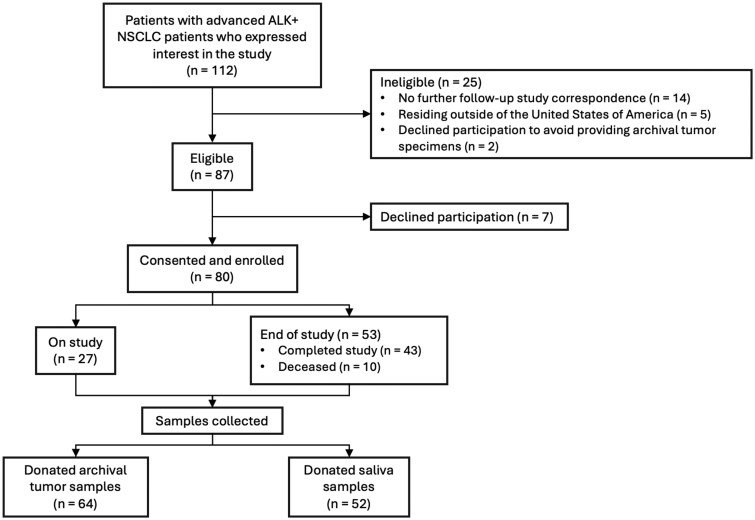
CONSORT diagram for the ENIGMA+ study.

Between December 2021 and September 2024, a total of 112 patients with advanced ALK+ NSCLC expressed interest in participating in the study, of whom 87 were determined to be eligible (Figure 1). Patients were deemed ineligible if they did not respond to further study correspondence (*n* = 14), resided outside of the United States (*n* = 5), or ultimately declined to participate to avoid providing archival tumor specimens (*n* = 2). Of the 87 eligible patients, 80 completed the remote consent process and were ultimately enrolled, constituting a rate of successful remote consent among interested and eligible patients of 92%.

Within the study cohort, the success rate for remote medical record collection was 100%. Archival tumor tissue and saliva samples were successfully collected from 80% and 65% of the enrolled patients, respectively. As of the data cut-off, 53 (66%) patients had completed the study, with follow-up visits ongoing for the remaining 27 (34%) patients. Of the 53 patients who completed the study, 81% (*n* = 43) had fulfilled all study requirements and follow-up timepoints, while 19% patients (*n* = 10) had died during the study timeframe.

### Patient demographics

**Figure 2.**
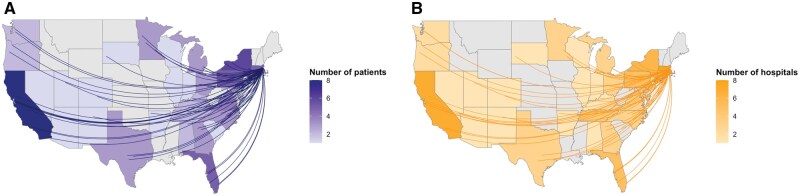
Geographic distribution of primary residence and treating hospitals for patients enrolled to ENIGMA+. Heatmaps display (A) the primary residence of enrolled patients, with states color-scaled based on the number of patients residing in each state and (B) the distribution of primary treating hospitals for enrolled patients, with states color-scaled based on the number of primary hospitals within those states. States with no patient involvement are shaded in gray.

The vast majority of enrolled patients (94%, *n* = 75) resided outside of Massachusetts (location of the study center), and 72 (90%) patients received their primary oncologic care outside Massachusetts (Figure 2). In total, 31 states of residence were represented by the study cohort. The most common states of patient residence were California (10%, *n* = 8), New York (8%, *n* = 6), Florida (6%, *n* = 5), Massachusetts (6%, *n* = 5), and Pennsylvania (6%, *n* = 5) (Figure 2A). The most common states for primary treating hospitals were Massachusetts (10%, *n* = 8), California (9%, *n* = 7), Florida (6%, *n* = 5), and Pennsylvania (6%, *n* = 5) (Figure 2B). Of the cohort, 49 (61%) received their primary oncologic care at academic medical centers/hospitals whereas the remainder did not (Table 1).

**Table 1.** Demographics and baseline characteristics of the ENIGMA+ study cohort (*n* = 80).

Characteristic	*N*	%
**Age at study enrollment, median (range)**	56 (30-78)	
**Age at diagnosis of advanced NSCLC, median (range)**	51 (29-68)	
**Sex**		
Female	54	68
Male	26	33
**Ethnicity**		
Non-Hispanic/Latino	42	53
Hispanic/Latino	1	1
Unknown/Other	37	46
**Race**		
White	32	40
Asian	8	10
Hispanic/Latino	1	1
Unknown	39	49
**Treating medical centers/hospitals**		
Community-based	31	39
Academic-based	49	61
**Smoking history**		
Never	60	75
Former	8	10
Unknown	12	15
**Histology**		
Adenocarcinoma	78	98
Squamous cell carcinoma	2	3
**Method of ALK detection**		
NGS	55	69
FISH	27	34
IHC	14	18
***ALK* fusion partner**		
*EML4*	51	64
Others	2	3
Unknown	28	35
***EML4-ALK* fusion variants** ^a^		
Variant 1	23	45
Variant 3a/b	8	16
Other variants	7	14
Unknown variants	13	25

Abbreviations: FISH, fluorescence in-situ hybridization; IHC, immunohistochemistry; NSCLC, non–small cell lung cancer; NGS, next-generation sequencing.

a Percentages represent the proportion of patients with known *EML4* fusion partner.

The median age of patients at study enrollment was 56 years (range: 30-78 years), and the median age at diagnosis of advanced lung cancer was 51 years (range: 29-86 years). Fifty-four (68%) patients were women, and 60 (75%) had no history of smoking. Most patients (98%; *n* = 78) had adenocarcinoma histology (Table 1). Seventy-eight patients had advanced lung cancer at initial diagnosis, while 2 patients were initially diagnosed with early-stage lung cancer and subsequently had disease recurrence or progression.

The years of diagnosis for advanced ALK+ lung cancer ranged from 2011 to 2022, with a median diagnosis year of 2019. Presence of *ALK* fusion at diagnosis was determined using next-generation sequencing (NGS) in 55 (69%) patients, fluorescence in situ hybridization (FISH) in 27 (34%), and immunohistochemistry (IHC) in 14 (18%) (Table 1). Of the 52 patients with a known *ALK* fusion partner, the most common fusion partner was *EML4* in 51 patients (Table 1). Other fusion partners included *LTBP1* (*n* = 1), along with co-expressions of fusion partners *SLC8A1* and *EML4* (*n* = 1). Fusion partner was unknown/not specified for 28 patients (Table 1). Of the 51 patients with *EML4-ALK* fusion-positive disease, the most common variants were variant 1 (45%; *n* = 23) and variant 3a/b (16%; *n* = 8) (Table 1).

At the time of diagnosis of advanced ALK+ lung cancer, 22 (28%) patients presented with intrathoracic disease only and 56 (70%) patients with extrathoracic and intrathoracic metastases at baseline, with a median of 3 metastatic sites of disease (range: 2-6). The most common sites of metastatic involvement were bone (*n* = 36), pleura (*n* = 34), and brain (*n* = 27).

### Real-world treatment patterns and outcomes

**Figure 3.**
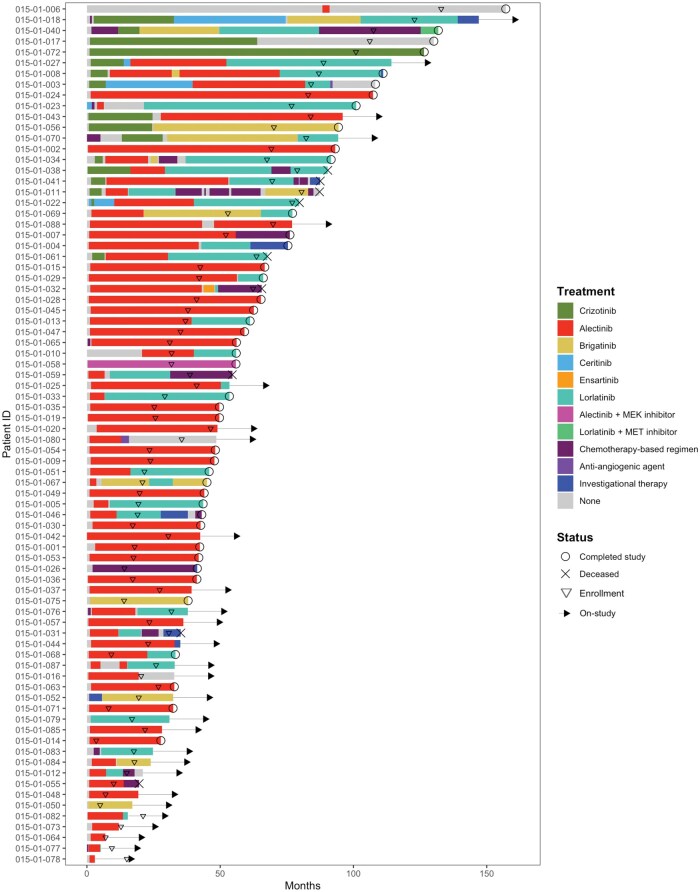
Treatment patterns of patients with ALK+ NSCLC. The swimmer plots demonstrate the treatment histories of patients enrolled in ENIGMA+. Colors indicate distinct systemic therapies. Day 0 is defined as the date of diagnosis of advanced or metastatic disease. Key events are denoted as follows: ENIGMA+ study enrollment (“▽”), study completion (“O”), deceased (“X”), and ongoing participation (“→”).

Treatment regimens received by the study cohort included ALK TKIs (crizotinib, alectinib, brigatinib, ceritinib, ensartinib, and lorlatinib), chemotherapy, anti-angiogenic agents, investigational therapies, and combination therapies (Figure 3).

**Figure 4.**
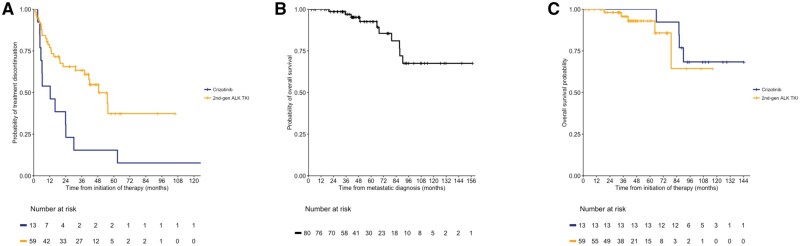
Durations of therapy and overall survival. The Kaplan-Meier survival curves demonstrate the (A) treatment duration on the first-line crizotinib or first-line second-generation ALK TKI (day 0 defined as the date of relevant treatment initiation), (B) overall survival of the study cohort (day 0 defined as the date of diagnosis of advanced or metastatic disease), and (C) overall survival in patients treated with crizotinib versus second-generation ALK TKI as first-line therapy (day 0 defined as the date of relevant treatment initiation).

Crizotinib was initiated as the first-line therapy for 13 (16%) patients, with a median initiation year of 2015 (range: 2013-2017). Alectinib was the first-line therapy for 56 (70%) patients, with a median initiation year of 2020 (range: 2015-2022), reflecting the evolving treatment landscape for this disease. Additionally, 3 (4%) patients received chemotherapy as first-line treatment, with a median start year of 2015 (range: 2012-2021). The median duration of first-line crizotinib was 12 months (95% CI, 5.4-NR), while the median duration of first-line second-generation ALK TKIs was 49 months (95% CI, 41-NR) (Figure 4A). At the 5-year landmark, 15% (95% CI, 4.3-55) of patients treated with crizotinib had experienced either disease progression or a treatment switch, compared to 37% (95% CI, 22-62) of those treated with second-generation ALK TKIs.

The second- or later-line therapies received by patients differed based on the initial treatment received (Figure S1). Among the 13 patients who received first-line crizotinib, the most common second-line treatments were alectinib (*n* = 8) and ceritinib (*n* = 3) (Figure S1A). Of the 56 patients who received first-line alectinib, 50% of the patients had not changed therapies since initiation till the most recent -up visit before data cut-off. Fifteen (27%) transitioned to lorlatinib as second-line therapy, 3 (5%) started chemotherapy-based regimens, and 3 (5%) started brigatinib (Figure S1B). Sequential therapies following chemotherapy included crizotinib (*n* = 1), brigatinib (*n* = 1), and investigational therapy (*n* = 1) (Figure S1C).

OS outcomes were immature as of this analysis. The overall 5-year survival rate for patients enrolled in the study was 93% (95% CI, 86-100), with the median survival not reached (Figure 4B). The median duration of follow-up for OS was 20.5 months (95% CI, 20.3-20.9). The 5-year OS rate was 93% (95% CI, 85-100) in patients treated with second-generation ALK TKIs as their initial therapy and 100% (95% CI, 100-100) in those treated with crizotinib as initial therapy (Figure 4C). Among the patients treated with first-line crizotinib, 92% (*n* = 12) continued with second-line second-generation ALK TKIs, including alectinib (*n* = 8), ceritinib (*n* = 3), and brigatinib (*n* = 1) (Figure S1A).

### Archival tissue and saliva collection

Sixty-four patients (80%) provided a total of 103 biopsy specimens. A detailed summary of the tumor samples and relevant treatments received prior to the biopsies is provided in Figure S2. Seventy treatment-naïve tumor biopsy specimens were collected from 51 patients. A total of 33 ALK TKI-resistant tumor samples were collected from 35 patients. Ten treatment-resistant samples were collected at disease progression on the patients’ first-line ALK TKI, including crizotinib (*n* = 1) and second-generation ALK TKI (*n* = 9). Seventeen lorlatinib-resistant (all with lorlatinib administered as later-line therapy) samples were obtained from 14 patients. Sixteen patients contributed serial biopsy samples spanning more than one treatment timepoint (eg, treatment-naïve and treatment-resistant, or at resistance to distinct TKIs). The most common anatomic sites biopsied and collected for archive included lung (specimen: *n* = 51), lymph nodes (specimen: *n* = 35), and brain (specimen: *n* = 5). Matched saliva samples were obtained from 52 patients.

### Molecular profiling

Of the study cohort, 55 patients had local NGS performed at the time of the initial diagnosis of advanced NSCLC with results reported, and 18 had NGS performed at the time of resistance to ALK TKI(s). The NGS platforms used included FoundationOne (*n* = 24), GuardantOne (*n* = 17), and Tempus (*n* = 8), among others.

**Figure 5.**
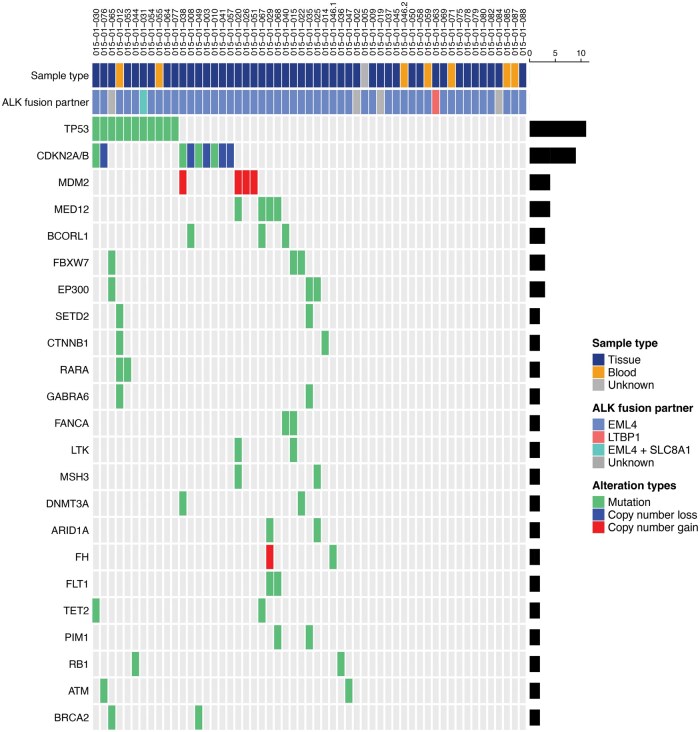
Genomic profiling of ALK+ NSCLC at initial diagnosis. The heatmap demonstrates gene alterations identified upon next-generation sequencing (NGS) of the initial diagnostic biopsies, including *ALK* fusion variants and co-alterations (including variants of unknown significance). Only those genes found to be altered in at least two patients are included in the heatmap.

Within the treatment-naïve cohort, the most frequently detected co-alteration on NGS testing (tissue: *n* = 48; liquid: *n* = 7) was *TP53* mutations (*n* = 10), followed by *CDKN2A/B* variants (*n* = 9). Additional commonly altered genes include *MDM2* (*n* = 4), *MED12* (*n* = 4), *BCORL1* (*n* = 3), *FBXW7* (*n* = 3), and *EP300* (*n* = 3) (Figure 5).

**Figure 6.**
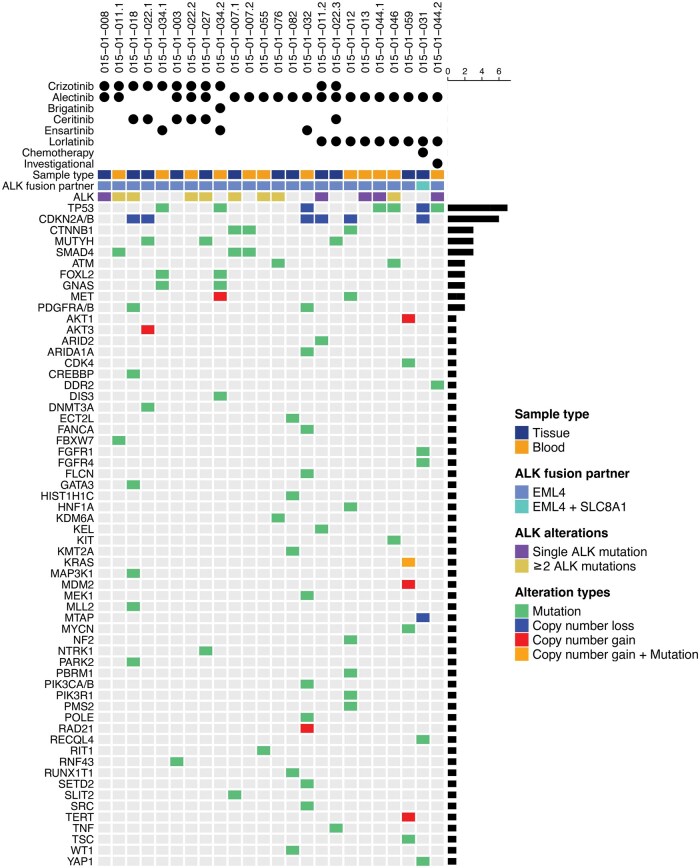
Genomic profiling of ALK+ NSCLC at ALK inhibitor resistance. The heatmap indicates gene alterations identified upon NGS of post-progression biopsies. Gene alterations were excluded if predicted to be benign according to the OncoKB database.^19^^,^^20^ Systemic therapies received prior to the resistance biopsies are indicated above the heatmap.

Among the 18 patients who had treatment-resistant cases analyzed by local NGS testing (tissue: *n* = 12; liquid: *n* = 12), all had progressed on second-generation ALK TKI(s), with 61% (*n* = 11) also having progressed on crizotinib and 50% (*n* = 9) having progressed on lorlatinib. The most commonly used second-generation ALK TKIs in this cohort were alectinib (*n* = 15) and ceritinib (*n* = 4). *ALK* resistance mutations were identified in 13 (54%) treatment-resistant biopsies that underwent NGS. Among 6 samples resistant to at least one second-generation ALK inhibitor (alectinib only: *n* = 5, alectinib and ceritinib: *n* = 1), 3 (50%) had a single *ALK* mutation (G1202R: *n* = 2, V1180L: *n* = 1). Among 9 samples resistant to lorlatinib and at least one second-generation ALK TKI, one had a single *ALK* mutation (G1202R: *n* = 1), and 4 had compound *ALK* mutations. One sample resistant to investigational therapy and prior lorlatinib and alectinib harbored a compound *ALK* mutation (G1202R + F1174V + C1156Y). Other common co-altered genes across ALK TKI-resistant biopsies included *TP53* (*n* = 7), *CDKN2A/B* (*n* = 6), *CTNNB1* (*n* = 3), *MUTYH* (*n* = 3), and *SMAD4* (*n* = 3) (Figure 6).

The distribution of PD-L1 expression levels was also assessed. Among 46 treatment-naïve samples tested locally for PD-L1 expression, 14 (30%) had a high-level tumor proportion score (TPS ≥50%), 17 (37%) intermediate level (1%-49%), and 15 (33%) low level (<1%). Of 11 ALK TKI-resistant cases, 3 (27%) had high-level, 4 (36%) had intermediate-level, and 4 (36%) had low-level expression of PD-L1.

## Discussion

This study demonstrated the feasibility of a fully remote, nationwide decentralized clinical research initiative that enhanced the access of patients with advanced ALK+ NSCLC to study participation. The findings highlighted the high success rate of remote consenting, underscoring the effectiveness of virtual engagement. Medical record collection achieved a 100% success rate, and 80% of participants were able to contribute archival tumor specimens for further correlative analyses.

Notably, the ENIGMA+ study cohort was geographically diverse, with 94% of participants residing outside the study site and receiving oncologic care across different US states. This decentralized research approach, therefore, facilitated a broader representation of patients with ALK+ NSCLC, the overarching goal of our initiative. Historically, translational and clinical research endeavors, including in ALK+ NSCLC, have primarily been centered at a single or a select few large academic centers, subject to sample bias and concerns regarding the generalizability of the knowledge thus generated.^18, 21–23^ The ENIGMA+ framework surpasses these limitations and, in parallel, begins to tackle aspects of longstanding disparities in clinical research participation.^24^ Participants in the ENIGMA+ study could avoid devoting their energy, financial, and time resources to travel to a centralized research site physically. This approach can potentially enhance accessibility and inclusivity, particularly for those individuals facing physical or health-related, financial, or psychosocial barriers to participation.^15^^,^^25^^,^^26^ Indeed, a LUNGevity survey of 137 patients revealed that among patients with metastatic NSCLC, approximately 89% are unable to physically travel to a referral center for a second opinion due to reasons including lack of insurance coverage for referrals (56%), financial barriers to travel (57%), or inability to take time away from work (47%) (personal communications with Dr Upal Roy, LUNGevity Foundation). This is likely an underestimate due to underreporting, especially from the communities of patients who are not being represented in these surveys.^27^^,^^28^ Such patients would likely not have access to any research studies or clinical trials physically offered at the referral center but could be represented by studies like ENIGMA+.

Remote consenting has been in development since the 2010s.^29^ However, its systemic integration into clinical research did not gain momentum until the COVID-19 pandemic, when in-person trials were visibly and jarringly disrupted.^30^ Since then, secure online platforms have streamlined processes for cancer research as well as post-operative care and chronic disease management.^31–33^ Despite these advancements, standardized nationwide systems for remote consent, real-time clinical data collection, and biospecimen acquisition remain underdeveloped. This study addresses a critical gap by presenting a scalable model for decentralized oncology research that seamlessly integrates remote consent and follow-ups. Its fully remote design, utilizing a patient-facing website and REDCap data collection infrastructure, enabled structured and patient-centered study introduction and discussion, informed consent, and follow-ups at 6, 12, and 24 months, facilitating longitudinal collection of real-world treatment and genomic data.

Treatment data from the ENIGMA+ cohort reflected real-world prescribing patterns consistent with the evolving NCCN guidelines for ALK+ NSCLC.^34^ Most patients received next-generation ALK TKIs, including alectinib, brigatinib, and lorlatinib, aligning with current standards of care. As the ENIGMA+ study was designed primarily as a feasibility and biospecimen collection initiative rather than a therapeutic trial, treatment outcomes should be interpreted in the context of a non-randomized, observational setting. Notably, OS outcomes were likely influenced by variability in the timing of study entry relative to initial diagnosis, and results were also immature as of data cut-off. The cohort’s treatment initiation dates, ranging from 2012 to 2022 (median 2020), mirror the evolving therapeutic landscape of ALK+ NSCLC during this period. As lorlatinib was more recently approved as a first-line therapy, only one participant had initiated treatment with lorlatinib upfront; however, it was commonly used as a subsequent-line therapy across the cohort.^4^^,^^35^

The development of resistance to ALK TKIs represents a major clinical issue that tempers the degree of therapeutic benefit for patients. Molecular analyses of post-progression biopsies have provided fundamental insights into resistance mechanisms to ALK TKIs, shaping the development of new therapies.^36–39^ However, historically, translational research on ALK resistance has been limited to individual institutions, restricting generalizability. One of the main goals of ENIGMA+ was to establish a large clinically annotated biospecimen repository derived from a broader nationwide patient population. The initial findings demonstrate the feasibility of collecting single or multiple tumor biospecimens and matched saliva from patients through a remote decentralized research infrastructure. Preliminary analyses of the available NGS profiles identified commonly co-altered genes involving *TP53*, *CDKN2A*/B, and others across ALK+ tumors, concordant with prior reports.^40^^,^^41^ The ENIGMA+ accrual and assessments continue. The next phase will include the launch of translational studies of the cohort biospecimens, including archival tissue and saliva, aimed at elucidating the genomic and immune biology of ALK+ tumors. These studies should help elucidate mechanisms underlying resistance vs exceptional responses to ALK TKIs.

The study has important limitations. First, data collection relied on patient-reported information and available external medical records, introducing potential data quality and accuracy variability. On the other hand, the study promoted direct remote patient participation throughout follow-ups, emphasizing the patient-centered aspect of its design. Second, while the demographic profile of the cohort overall was representative of patients with ALK+ NSCLC—primarily young individuals with no prior smoking history—and despite the geographic diversity achieved, racial and ethnic diversity remained limited, reflecting the limitations in study sample size. Furthermore, although the cohort represented 31 distinct states, specific regions, such as the Midwestern and Mountain states, remained underrepresented. One factor that may have contributed is the study’s primary reliance on outreach through social media platforms, which may have led to preferential reach toward patient subgroups more familiar with, or with easier access to, technology. Future research should address barriers to research access and enrollment among underrepresented populations to continue tackling the existing trial participation gaps. Third, archival tissue biospecimens were unable to be collected from 20% of patients, which may be due to various factors, including the exhaustion of tissue from clinical testing or other trial use and insufficient tissue even at diagnosis. In addition, any archival tissue or saliva collected after the data cut-off date for this initial analysis was not captured. Despite these limitations, the majority of patients were able to contribute their biospecimens toward the initiative, and biospecimen collection efforts continue. Finally, while conceptually the initial remote consent infrastructure could be adopted for therapeutic intervention trials, the unique challenges and complexities of therapeutic intervention trials (such as for safety monitoring, drug administration, etc.) must be acknowledged.

The expansion of remote data and biospecimen collection has the potential to revolutionize translational oncology research by overcoming some of the traditional barriers to study participation. If implemented on a larger scale, this study design could enable the creation of clinical databases and biospecimen repositories that more adequately represent diverse patient populations and thereby yield more generalizable and equitable knowledge on cancer biology and clinical trajectories. Such efforts could ultimately inform optimal precision oncology paradigms and improve patient outcomes.

## Supplementary Material

oyaf217_Supplementary_Data

## Data Availability

Data will be shared on request to corresponding authors.
