# Supplementary material for: ENIGMA+: a national, decentralized, remote consent study for clinical data and biospecimen collection in patients with ALK-positive advanced NSCLC
Source: Oncologist. 2025 Jul 17;30(9):oyaf217. doi: 10.1093/oncolo/oyaf217 (PMC12404294; doi:10.1093/oncolo/oyaf217)
Supplement: oyaf217_Supplementary_Data [file oyaf217_supplementary_data.zip › 041824_ENIGMA-Oncologist_sublegend.docx]

**SUPPLEMENTAL FIGURES**

**Supplementary** **Figure 1. Sequential treatment patterns according to the first-line therapy.** The Sankey plots demonstrate sequential therapies received by the ENIGMA+ study cohort, according to the first-line treatment: (A) crizotinib (n = 13), (B) alectinib (n = 56), or (C) chemotherapy (n = 3). The asterisk (*) Indicates therapy was held due to treatment toxicities before restarting. The symbol (**) denotes that therapies were given in combination with additional systemic treatments.

**Supplementary** **Figure 2. Archival tumor sample collections stratified by prior therapies. The number of** archival tumor samples collected through the ENIGMA+ study are indicated, stratified based on treatments received prior to the biopsies. Abbreviations: gen, generation; TKI, tyrosine kinase inhibitor.
