# Supplementary figures and images for: ENIGMA+: a national, decentralized, remote consent study for clinical data and biospecimen collection in patients with ALK-positive advanced NSCLC
Source: Oncologist. 2025 Jul 17;30(9):oyaf217. doi: 10.1093/oncolo/oyaf217 (PMC12404294; doi:10.1093/oncolo/oyaf217)

# Supplemental Figure 1

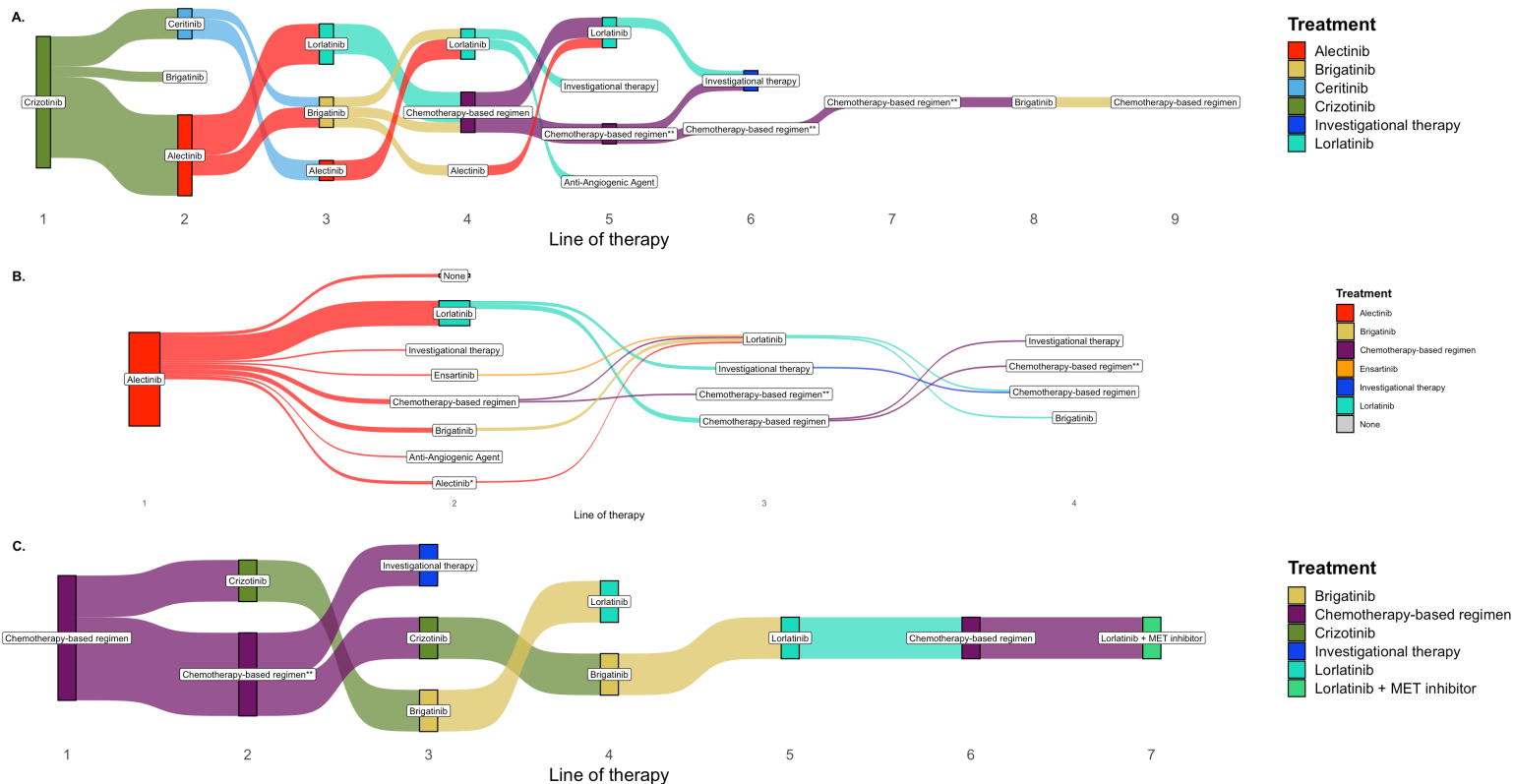

Supplemental Figure 2

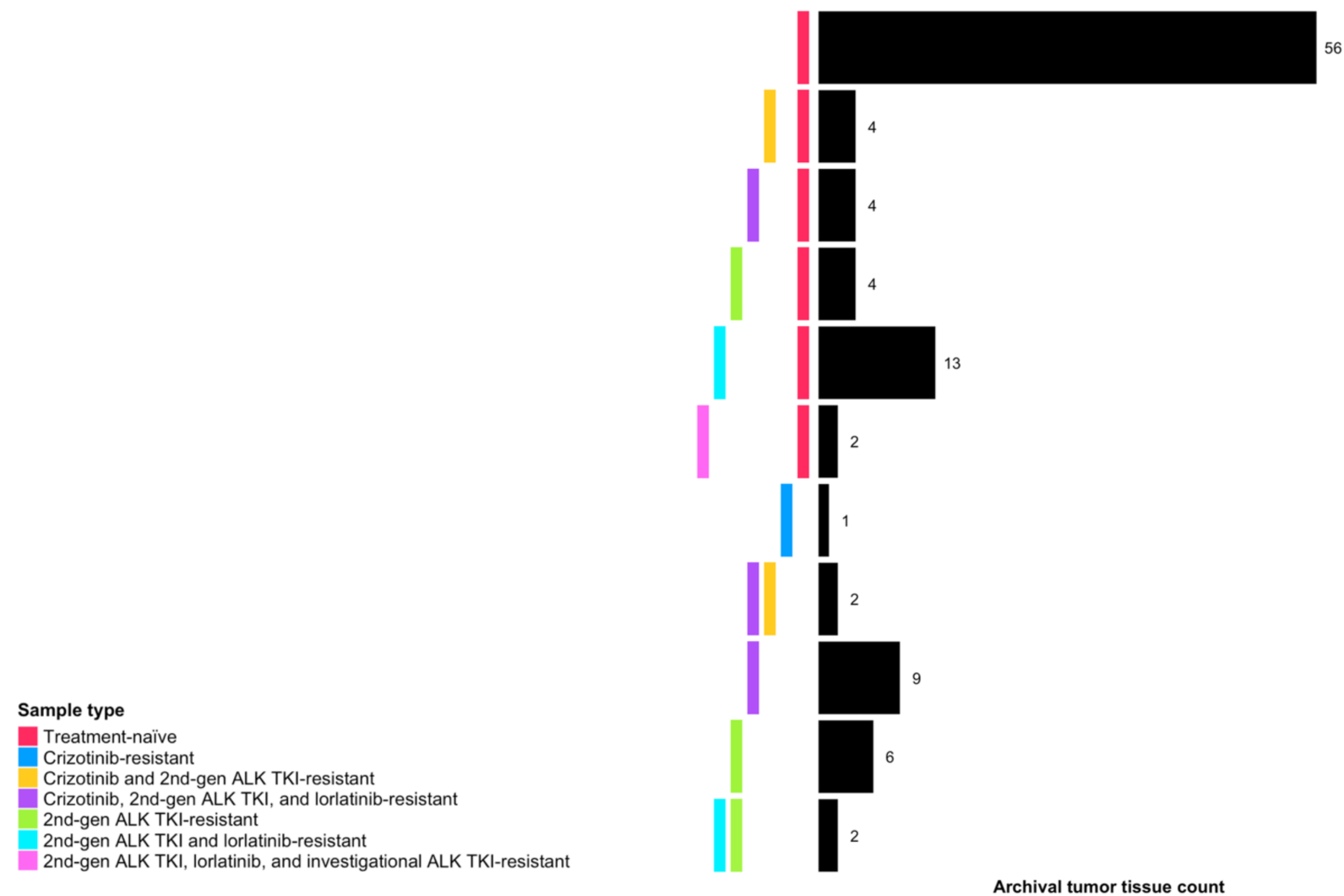

Supplement: oyaf217_Supplementary_Data [file oyaf217_supplementary_data.zip › Supplemental Figure 1.pdf]
